# 

**Published:** 1891-07

**Authors:** 


### BOOKS AND PAMPHLETS RECEIVED.

A Dermatological Bibliography, compiled by George Thomas Jackson, M. D., New York, 1891.

Bulletins of the Illinois State Laboratory of Natural History, Campaign, Illinois. Vol iii. Articles i-xi.

Notes on Some Illinois Microsgasters; with Descriptions of New Species. By Clarence M. Weed, M. Sc.

Jassidae of Illinois. Part 1. By Charles W. Woodworth, M. S.

On the Parasites of the Lesser Apple Leaf-Roller, *Teras minuta* (Robs). By Clarence M. Weed.

On the Anatomy and Histology of a New Earthworm (*Diplocardia communis*, gen. et sp. nov.). By H. German.

A Descriptive Catalogue of the Phalangiinœ of Illinois. By Clarence M. Weed, M. Sc.

A Partial Bibliography of the Phalangiinœ of North America. By Clarence M. Weed, M. Sc.

On an American Earthworm of the Family Phreoryctidœ. By S. A. Forbes.

An American Terrestrial Leech. By S. A. Forbes.

A preliminary report of the Animals of the Mississippi Bottoms, near Quincy, Illinois, in August, 1888. Part 1.

Notes on Illinois Reptiles and Amphibians, including several species not before recorded from the Northern States. By H. Garman.

Descriptions of new Cynipidœ in the Collection of the Illinois State Laboratory of Natural History. By C. P. Gillette, of the Iowa Experiment Station.

Some Comparative Osteological Notes on the North American Kites. By R. W. Shufeldt, C.M.Z.S. Rep., from the Ibis. April 1891.

Bulletin de la Société Vandoise des Sciences Naturelles. Lausanne. 1891.

Johns Hopkins University Circulars. 1891.

Proceedings of the Linnean Society of New South Wales. Vol. 4. p. 2-3. Sydney.

Abhandlungen Naturwissenschaftlichen Vereine Zu Bremen. Bd. xii, Heft 1. 1891.

Annual Report of the Zoölogical and Acclimatization Society of Victoria. 1891.

The Ottawa Naturalist. Vol. v. No. 3.

Transactions of the Canadian Institute. Vol. 1. p. 2. Toronto, 1891.

Annual Report of the Canadian Institute, Toronto 1891.

Archives Neerlandaises des Sciences Exactes et Naturelles, edited by J. Bosscha Tome, xxv. Lir 1. Harlem 1891.

Bollettino Scientifico. Paris 1891.

Anales de la Sociedad Cientifica Argentina, Buenos Aires 1891.

Bulleten de la Société Zoologique de France. Paris 1891.

Atti e Rendiconti della Academia Medico-Cherurgica di Perugia, 1891.

Bulletin Mensuel Société Linneenne du Nord de la France. Amiens, 1891.

